# Natural coumarins from *Murraya paniculata* as mixed-type inhibitors of cholinesterases: *In vitro* and *in silico* investigations

**DOI:** 10.3389/fphar.2023.1133809

**Published:** 2023-03-09

**Authors:** Asaad Khalid, Waqasuddin Khan, Komal Zia, Azizuddin, Waquar Ahsan, Hassan A. Alhazmi, Ashraf N. Abdalla, Asim Najmi, Andleeb Khan, Abdelhakim Bouyahya, Zaheer Ul-Haq, Ajmal Khan

**Affiliations:** ^1^ Substance Abuse and Toxicology Research Center, Jazan University, Jazan, Saudi Arabia; ^2^ Medicinal and Aromatic Plants and Traditional Medicine Research Institute, National Center for Research, Khartoum, Sudan; ^3^ Dr. Panjwani Center for Molecular Medicine and Drug Research, International Center for Chemical Sciences, University of Karachi, Karachi, Pakistan; ^4^ Department of Pediatrics and Child Health, The Aga Khan University, Karachi, Pakistan; ^5^ Department of Chemistry, Federal Urdu University of Arts, Science and Technology, Karachi, Pakistan; ^6^ Department of Pharmaceutical Chemistry and Pharmacognosy, College of Pharmacy, Jazan University, Jazan, Saudi Arabia; ^7^ Department of Pharmacology and Toxicology, College of Pharmacy, Umm Al-Qura University, Makkah, Saudi Arabia; ^8^ Department of Pharmacology and Toxicology, College of Pharmacy, Jazan University, Jazan, Saudi Arabia; ^9^ Laboratory of Human Pathologies Biology, Faculty of Sciences, Mohammed V University in Rabat, Rabat, Morocco; ^10^ Natural and Medical Sciences Research Center, University of Nizwa, Nizwa, Sultanate of Oman

**Keywords:** acetylcholinesterase, butyrylcholinesterase, natural coumarins, inhibition kinetics, molecular docking, MD simulation, ligand-enzyme interactions

## Abstract

Currently, acetylcholinesterase (AChE) inhibiting drugs in clinical use, such as tacrine, donepezil, rivastigmine, and galanthamine, are associated with serious side effects and short half-lives. In recent years, numerous phytochemicals have been identified as inhibitors of cholinesterases with potential applications in the management of Alzheimer’s disease (AD). In this study three natural coumarins, 2′-O-ethylmurrangatin (**1**), murranganone (**2**), and paniculatin (**3**) isolated previously by our group from the leaves of Murraya paniculata, were tested against the two cholinesterases (ChE) enzymes, AChE and butyrylcholinesterase (BChE) using *in vitro* assay. Molecular docking was performed to highlight the structural properties that contribute to the molecular recognition pattern in the inhibition of ChE and the structural differences resulting in the selectivity of these compounds toward AChE. Classical enzyme inhibition kinetics data suggested that compounds **2** and **3** were potent inhibitors of AChE and BChE, while **1** was found inactive against both enzymes. The findings from molecular docking studies revealed the competitive and non-competitive inhibition mechanisms of compounds **2** and **3** against both enzymes. Molecular docking and simulations have revealed that hydrogen bonding, mediated by ketone and hydroxyl functionalities in various positions, significantly contributes to the binding of the inhibitor to the receptor. According to MD simulation studies, the stability of the ligand-AChE complex for the most active compound (**3**) is found to be comparable to that of the widely used drug Tacrine. In addition, to evaluate the drug-likeness of compounds, *in silico* ADME evaluation was performed, and the compounds presented good ADME profiles. Data suggested that the coumarin nucleus having diverse side chains at the C-8 position can serve as a potential inhibitor of cholinesterases and can act as a lead to develop a new semisynthetic drug for the treatment of AD.

## 1 Introduction

Alzheimer’s disease (AD) is a neurodegenerative disorder that leads to a gradual loss of neuronal structure and function, causing cognitive decline and dementia (Kabir et al., 2021; Onoja et al., 2021; Khalid et al., 2022). The neurotransmitter Acetylcholine (ACh), which is essential for learning and memory processes, was the first neurotransmitter to be implicated in Alzheimer’s disease (Kaur et al., 2022; Hussain et al., 2023). The ChE family of enzymes consists of AChE, which hydrolyzes ACh in the cholinergic synapses, and BChE, which preferably hydrolyzes butyrylcholine (BCh), having an elusive function in the central nervous system (CNS). Both enzymes are considered potential targets for discovering mechanism-based inhibitors for the treatment of AD and related neurodegenerative disorders (Rampa et al., 2013; Karthika et al., 2022). The role of BChE in the normal, aging, and diseased brain is still unknown; however, it was found that the BChE existed in significantly higher quantities in Alzheimer’s plaques than in the plaques of age-related non-demented brains (Darvesh et al., 2012). Moreover, the inclusion of cymserine, a highly potent selective BChE inhibitor in the clinical trials for AD treatment, proved that BChE inhibition could be an essential tool for the treatment of AD and related dementias (Kamal et al., 2008). The structures of AChE and BChE are very close to each other. Overall, cholinesterases have α/β hydrolase protein fold, and the active sites of both cholinesterases are buried near the bottom of a deep gorge which consists of esteratic and π-cation subsites (Hotelier et al., 2004).

In AChE, the gorge is lined by 14 conserved aromatic amino acids, whereas in BChE, Leu286 and Val288 are positioned by replacing structurally equivalent AChE residues, Phe288 and Phe290, respectively, facilitating BChE to accommodate bulkier and non-polar ligands. At the opening of the gorge, an anionic site termed as peripheral anionic site (PAS) is present, which makes the diffusion of the substrate possible towards the active site (Fang et al., 2011). In contrast to AChE, BChE lacks Trp279 resulting in weaker binding of bisquaternary ligands at the PAS. The ChE is known to be inhibited by a wide variety of synthetic and natural compounds (Houghton et al., 2006; Murray et al., 2013), and previously, several natural ChE inhibitors isolated from various medicinal plants were reported by our group (Parveen et al., 2001; Atta-ur-Rahman et al., 2002; Atta-Ur-Rahman et al., 2003; Choudhary et al., 2006). The *in vitro* and *in silico* studies aimed to perform inhibition kinetics, Comparative Molecular Field Analysis (CoMFA), Comparative Molecular Similarity Indices (CoMSIA), ligand docking, and molecular dynamics (MD) simulation studies have also been conducted on these inhibitors (Wellenzohn et al., 2003; Wheeler, 2003; Zaheer et al., 2003).

**FIGURE 1:**
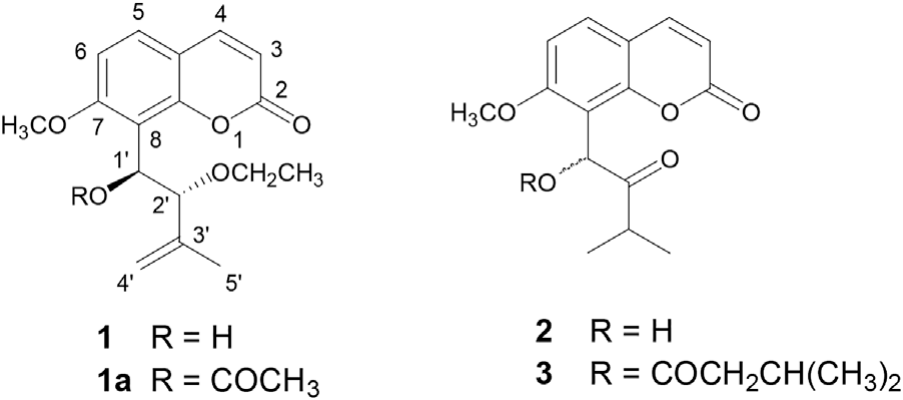
Strcutures of isolated coumarins from *Murraya paniculata* (Orange Jasmine) 2′-*O*-ethylmurrangatin (**1**), murranganone (**2**), and paniculatin (**3**).

Coumarins are one of the most important moieties that are present in a number of natural and synthetic drugs acting on various targets. Recently, coumarins have been explored for their potential as an important pharmacophore in the development of AChE inhibitors, and promising results were obtained (Abu-Aisheh et al., 2019; de Souza et al., 2016; Ekstrom et al., 2022; Sonu et al., 2019; Yusufzai et al., 2018). Previously, we reported the isolation and characterization of three 7-methoxy-8-substituted coumarins, namely, 2′-*O*-ethylmurrangatin (**1**), murranganone (**2**), and paniculatin (**3**) from *Murraya paniculata* (Orange Jasmine) (Figure 1) (Choudhary et al., 2002). *Murraya paniculata* has multiple traditional medicinal uses, including treatment for diarrhea, abdominal pain, and headaches, as well as anticonvulsant and antibacterial properties, and has been found to have antinociceptive, anti-inflammatory, antidiabetic, antimalarial, and antioxidant activities (Dosoky et al., 2016).

In this study, the efficacy and druggability of isolated coumarin derivatives were investigated against the two ChE enzymes using *in vitro* enzyme inhibition assay, molecular docking, MD simulation, and ADME profile evaluation studies.

## 2 Materials and methods

### 2.1 Materials

The three coumarins utilized in this study, i.e., 2′-O-ethylmurrangatin (**1**), murranganone (**2**), and paniculatin (**3**) were isolated from M. paniculata (Orange Jasmine) collected from Karachi, Pakistan as previously reported (Choudhary et al., 2002).

All the chemicals and solvents used in this study were purchased from Sigma Aldrich (Steinheim, Germany). The enzyme inhibition studies were performed on 96-well microtiter plates using a SpectraMax microplate spectrophotometer (Molecular Devices, CA, United States). AChE, BChE, acetylthiocholine, butyrylthiocholine, 5,5-dithiobis-2-nitro benzoic acid (DTNB), and Eserine (standard inhibitor) were also purchased from Sigma Aldrich (Steinheim, Germany). The enzyme kinetic study was performed using the EZ-Fit enzyme kinetics program (Perrella Scientific Inc., Amherst, United States). The software used for molecular docking was Molecular Operating Environment (MOE) version 2018 (Chemical Computing Group, Montreal, Canada). MD simulation study was performed using GROMACS version 2018 (University of Groningen, Netherlands), and ADME prediction was made using SwissADME (Swiss Institute of Bioinformatics, Lausanne, Switzerland).

### 2.2 *In vitro* enzyme inhibition assay

The AChE and BChE enzymes inhibition activity was measured *in vitro* by a modified spectrophotometric method developed by Ellman et al. (1961) with slight modifications. The test compounds were prepared fresh immediately before each experiment. Test compounds were added to 150 µl 0.1 mM phosphate buffer (pH 8.0), 200 µl methanol, and 20 µl of the test enzymes and were incubated at 25°C for 15 min. A sample with an equal amount of solvent (ethanol) in place of the test compounds served as a negative control. 10 μl of DTNB reagent was added to the samples, and the reaction was initiated by adding 10 μl acetylthiocholine (ATCh) or butyrylthiocholine (BTCh) for the estimation of AChE and BChE, respectively. All the experiments were conducted in triplicate. The hydrolysis of ATCh and BTCh was measured by assessing the formation of a yellow anion of 5-thio-2-nitrobenzoate at wavelength 412 nm due to the reaction of DTNB with thiocholine. The rate of enzyme inhibition was calculated as the change in optical density per minute (OD/min). The rate of enzymatic reaction was calculated using the extinction coefficient of 5-thio-2-nitrobenzoate by the following equation:

$$Rate\;\left(moles/L/\min\right)=\frac{change\;in\;absorbance}{\varepsilon_{TNB}}$$

where ε_TNB_ = molar extinction coefficient of 5-thio-2-nitrobenzoate = 13.6 × 10^3^ at 412 nm (Ellman, 1958).

### 2.3 IC_50_ estimation

The IC_50_ values were determined spectrophotometrically by measuring the concentrations of test compounds that inhibited the hydrolysis of substrates ATCh or BTCh, by 50%. The enzyme inhibition activities of serially diluted concentrations of test compounds were measured, and the IC_50_ values were calculated using EZ-Fit Enzyme Kinetics Program.

### 2.4 Enzyme kinetics and measurement of the dissociation constant

The dissociation constant (*Ki*) values, which signified the dissociation of the enzyme-inhibitor complex into free enzyme and inhibitor, were also determined. The *Ki* values were calculated by the interpretation of the Dixon plot (Cheng and Prusoff, 1973; Fukushima et al., 2002), the Lineweaver-Burk plot (Fjellstedt and Schlesselman, 1977), and its secondary re-plots by using initial velocities. These velocities were obtained over a range of substrate concentrations between 0.1 and 0.4 mM for ATCh and 0.05 and 0.2 mM for BTCh. The assay conditions for measurement of the residual activities of all inhibitors were identical to the aforementioned spectrophotometric assay procedure, except that the fixed concentrations of inhibiting compounds were used in the assay medium. Assays were conducted in triplicate at each concentration of the inhibitor.

### 2.5 Determination of inhibition pattern

The inhibition patterns of tested coumarins were determined by the graphical views of Dixon plots, Lineweaver-Burk plots, and their secondary re-plots. Graphs were plotted using the GraFit 7.0 curve fitting program (Erithacus Software Ltd., West Sussex, United Kingdom) (Yamada et al., 2017). The linear regression analysis values, correlation coefficient, slope, intercept, and standard errors were obtained using the same software. Two different methods were applied to monitor the effects of the test sample on both Michaelis constant (*K*_*m*_) and maximum velocity (*V*_max_) values. This was done first by plotting the reciprocal of the reaction rate against the reciprocal of the substrate concentration as the Lineweaver-Burk plot (Frère et al., 1983); and secondly by the Dixon plot in which the reciprocal of rates of reaction were plotted against the concentrations of the inhibitor (Yao et al., 2012). The secondary re-plots of the Lineweaver-Burk were also constructed in two ways; firstly, the reciprocal of apparent *V*_*max*_ (*1/V*_*maxapp*_) values were determined at each intersection point for every inhibitor concentration line on the *y*-axis of the Lineweaver-Burk plot and then re-plotted against different concentrations of the respective inhibitor. Secondly, in the non-competitive and linear mixed-type inhibitions, the slope of each line of inhibitor concentration on the Lineweaver-Burk plot was plotted against inhibitor concentrations. The secondary re-plot of the Dixon plot was constructed as the slope of each line of substrate concentration in the original Dixon plot against the reciprocals of substrate concentrations.

### 2.6 Molecular docking studies

Molecular docking studies were performed to get insights into the binding pattern of the natural coumarins against AChE and BChE enzymes. All the tested coumarins were sketched in the MOE program (Vilar et al., 2008) using a builder module and subjected to structure correction and protonation followed by energy minimization using an MMFF94 force field (Sulimov et al., 2017). Crystal structure of Tacrine in complex with AChE and BChE were retrieved from the Protein Data Bank (PDB ID: 1ACJ and 4BDS, respectively) (Harel et al., 1993; Nachon et al., 2013). All the water molecules were removed except the conserved water molecules, and hydrogen atoms were added for both proteins.

Crystal structures of both enzymes were minimized using an Amber99 force field (Wang and Wolf, 2004) prior to docking. Active aromatic gorge served as grid spacing during docking, and 30 poses were generated for all three coumarins. Triangular Matcher was used as placement method with London dG and GBVI/WSA dG as scoring and re-scoring functions, respectively. The binding patterns of coumarins in the active aromatic gorge of AChE and BChE were analyzed using Chimera.

### 2.7 Molecular dynamics (MD) simulation studies

The MD simulation studies were performed for AChE in complex with paniculatin **(3)** and the standard drug tacrine using GROMACS (Berendsen et al., 2005). Automated Topology Builder (ATB) was used to generate the topologies of test compounds, and the topology for the receptor was processed using GROMOS96 54a7 force field (Schmid et al., 2011). The cubic box was generated, the protein was placed at a distance of 1.0 nm from the box edge, and the systems were solvated using the SPC water model with periodic boundary conditions. Sodium ions were added to neutralize the systems. For energy minimization, the steepest descent algorithm was used, and the minimized systems were equilibrated in NVT and NPT ensemble for 100 ps by using Berendsen barostat and thermostat algorithms. For constraining the bond length and calculation of electrostatic long-range interactions, LINCS algorithm, and PME (particle mesh ewald) method were applied, respectively. The working conditions were set at 298.1 K temperature, 1 atm pressure, and 2.0 fs time per step. Finally, the MD production of 100 ns was run for the equilibrated systems. Root mean square deviation (RMSD) was analyzed to determine the stability of protein-ligand complexes.

### 2.8 ADME analysis

The ADME profiles of test compounds were predicted using a web-based server SwissADME (DeLano, 2002; Muhammad et al., 2021). The SMILES notations of all the three compounds were generated and subjected to the webserver for the prediction of physiochemical and pharmacokinetic properties. BOILED-Egg predictive model was applied to predict and estimate the probability of gastrointestinal absorption and permeation to brain.

## 3 Results and discussion

### 3.1 Cholinesterase inhibition activity

Compounds (**1–3**) were subjected to *in vitro* inhibitory activity assessment against AChE and BChE enzymes using the standard method. Experimentally, IC_50_ values (Table 1) showed paniculatin (**3**) was the most active against AChE (IC_50_ = 31.6) followed by murranganone (**2**) (IC_50_ = 79.1), while that 2′-O-ethylmurrangatin (**1**) was not active at 1 mM conc. Murranganone (**2**) was the most active compound against BChE (IC_50_ = 74.3 µM).

**TABLE 1:** The kinetics data for cholinesterase enzyme inhibition by the tested coumarins (**1**–**3**).

Compound	AChE			BChE		
	IC_50_ ^a^ (µM)	*K*_*i*_ ^b^ (µM)	Type of inhibition	IC_50_ (µM)	*K*_*i*_ ^b^ (µM)	Type of inhibition
2′-*O*-Ethylmurrangatin (**1**)	No activity upto 1 mM	ND^c^	ND	842.8 ± 13.0	ND	ND
Murranganone (**2**)	79.1 ± 2.02	50 ± 0.58	NC^d^	74.3 ± 0.75	22 ± 0.44	LM^e^
Paniculatin (**3**)	31.6 ± 0.03	43 ± 0.65	LM	>100	22.0 ± 0.60	LM
Tacrine	0.021 ± 0.002	0.23 ± 0.02	MT^f^	0.051 ± 0.005	0.025 ± 0.003	MT
Galanthamine	0.45 ± 0.02	0.19 ± 0.01	MT	39.1 ± 0.032	32 ± 0.33	NC

^a^ *K*_*i*_ and IC_50_ values are expressed as (mean ± SEM), where SEM, is the standard mean error of 3 experiments.

^b^ *K*_*i*_ is the mean of four values calculated from Lineweaver-Burk plot, its secondary re-plots, and Dixon plot.

^c^ ND, Not determined.

^d^ NC, non-competitive.

^e^ LM, linear mixed.

^f^ MT, mixed type.

Classical inhibition kinetics was used to investigate the mode of inhibition and to determine the *Ki* values. Results showed that paniculatin (**3**) was more potent against AChE, while murranganone (**2**) was more potent against BChE and both compounds did not show selectivity towards any of the two enzymes (Table 1). Paniculatin (**3**) was observed to be a mixed-type inhibitor of both enzymes (AChE and BChE), whereas murranganone (**2**) exhibited a pure non-competitive inhibition of AChE and mixed-type inhibition of BChE. The compound 2′-*O*-ethylmurrangatin (**1**) did not show any inhibition against AChE and a very weak activity against BChE, in concentrations up to 1 mM.

**FIGURE 2:**
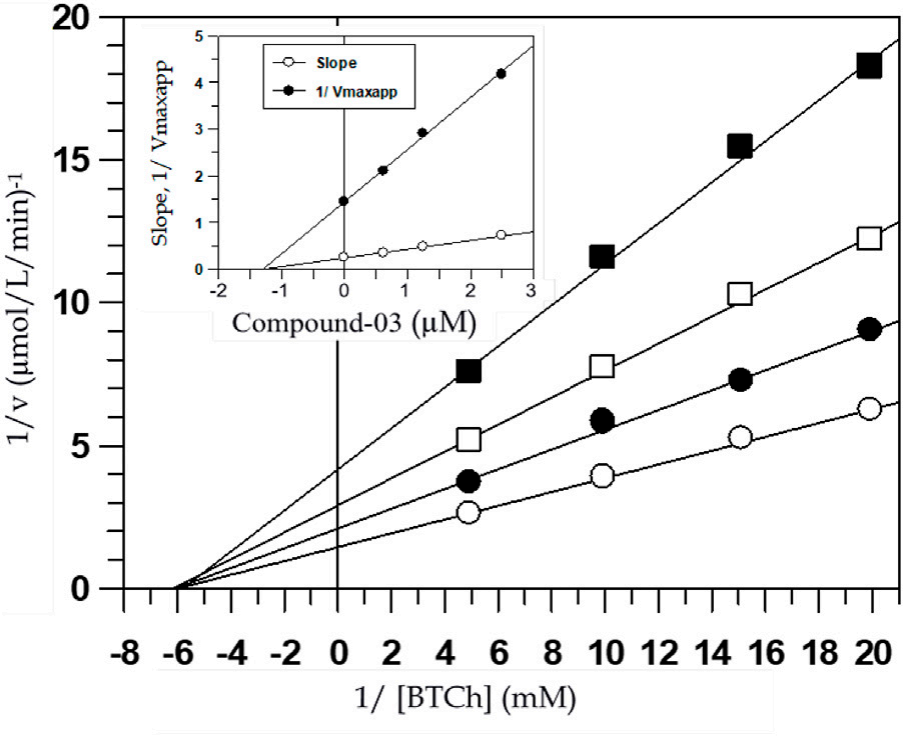
Lineweaver-Burk plot of reciprocals of the initial velocities versus reciprocals of BTCh in the absence (○) and presence of 1.25 µM (●), 2.5 µM (□) and 5 µM (■) of compound-03 against BChE. Inside frame shows the secondary replots of the Lineweaver-Burk plot: 1/V_maxapp_ or Slope versus various concentrations of inhibitor, the correlation coefficient for all the lines of all the graphs was >0.99, each point in all the graphs represents the mean of three experiments.

The inhibition patterns of tested compounds were determined using Lineweaver-Burk plots. Plots of compounds (**2**) and (**3**) for AChE inhibition were observed to be linear and the lines intersected at a point different from x- and *y*-axis. The *K*_*i*_ values were determined from the secondary plots of slopes of Lineweaver-Burk plots drawn against the concentration of inhibitors and were found to be 50 ± 0.58 and 43 ± 0.65 µM for compounds (**2**) and (**3**), respectively for AChE inhibition (Figure 2). Similarly, plots of BChE inhibition by both the compounds were also found to be linear and not intersecting at x- or *y*-axis. The Ki values calculated from the secondary re-plots were found to be 22 ± 0.44 and 22.0 ± 0.60 µM for compounds (**2**) and (**3**), respectively. These results showed that both compounds (**2**) and (**3**) acted as mixed-type inhibitors of AChE and BChE enzymes.

Mixed-type inhibitors decrease both *V*_*max*_ values and the affinity of the substrate towards the enzyme. Figure 2 shows the graphical representation of steady-state kinetic analysis of AChE and BChE inhibition by compounds (**2**) and (**3**). Mixed-type inhibition showed by naturally occurring coumarins under study was found to be a combination of partially competitive and pure non-competitive inhibitions (Rahman et al., 2006). The pure non-competitive inhibition of AChE by compound (**2**) was envisaged from the ability of the compound to decrease the *V*_*max*_ value without affecting the affinity of the enzyme for the substrate (*K*_*m*_ values). The purity of the non-competitive inhibition was further confirmed by the linearity obtained in the secondary re-plots of Lineweaver-Burk plots. This indicated that the compound (**2**) could fit in the substrate-binding loci of these enzymes and bind to the enzyme-substrate complex at other allosteric site such as the peripheral site.

The primary coumarin nucleus did not seem to have much effect on the inhibitory activity as the compounds (**1–3**) were structurally similar in having the coumarin nucleus but diverse at the side chain at the C-8 position, which might have accounted for their activity. Therefore, the structural basis of inhibitors binding at the active site gorge of both cholinesterases was important to investigate. To test the selectivity of these compounds for cholinesterases, all the three natural coumarins (**1**–**3**) were screened for their inhibition activity against several other enzymes such as urease, acid phosphatase, β-glucuronidase, and α−glucosidase. All the compounds were found inactive at concentrations up to 1 mM against all aforementioned enzymes, which indicated their selectivity towards cholinesterase enzymes.

### 3.2 Molecular docking studies

**FIGURE 3:**
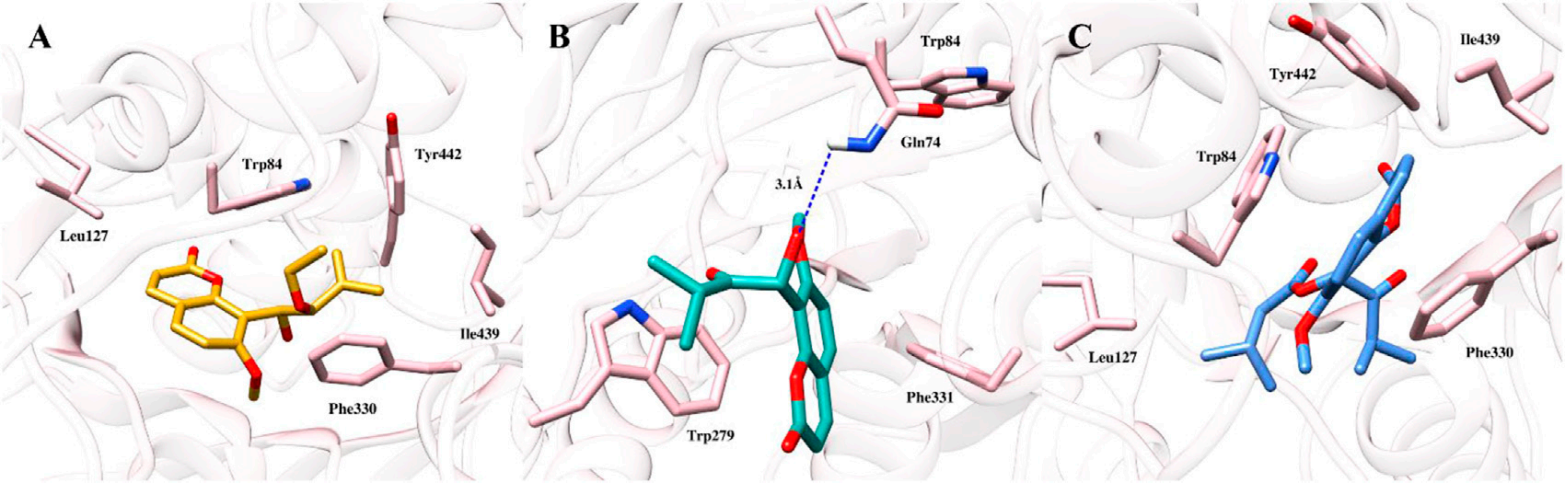
Docked pose of all the coumarin derivatives with AChE. **(A)** 2′-*O*-ethylmurrangatin (**1**) resided in the active aromatic gorge; **(B)** murranganone (**2**) fitted well in the peripheral anionic site of AChE and acted as non-competitive inhibitor; **(C)** paniculatin (**3**) resided in the anionic subsite of AChE.

To gain further insights into the binding modes and plausible mechanism of cholinesterase inhibition, molecular docking studies of all three compounds were carried out using crystal structure of AChE and BChE. The docking results revealed that all three compounds gained access to the deep aromatic gorge of AChE and BChE. The docking poses of compound (**1**) at the binding site of AChE suggested that the compound was unable to accommodate between Trp84 and Phe330 properly and showed the binding affinity of −3.28 kcal/mol. This might be attributed to the presence of unsaturated methylene (-enyl) group in the side chain at the C-3′ position as well as the absence of carbonyl group at C-2′ position which decreased the binding and made the compound inactive against AChE enzyme (Figure 3A). However, the ethoxy group at C-2′ position of compound (**1**) was observed to have hydrophobic interaction with Tyr442 residue.

Most of the docked poses of compound (**2**) showed that the compound bound to the peripheral anionic site at the aromatic gorge of AChE by interacting with Trp279 and Phe331 (Figure 3B) residues with the coumarin ring stacked between them. Similarly, isopropyl group present in the side chain at the carbonyl group-containing C-2′ position mediated alkyl-alkyl interaction with Trp279 which helped the compound to firmly bind to the AChE with the binding affinity of −4.07 kcal/mol. In addition, hydroxyl group at C-1′ position participated in the formation of a hydrogen bond with Gln74 which further improved the binding. The obtained results suggested that the compound (**2**) resided into the peripheral anionic site with good stability and showed non-competitive binding. Compound (**3**) was observed to be stacked in the binding site of AChE between the indole and phenyl rings of Trp84, Phe330 and Tyr442 amino acids with the highest binding affinity of −4.5 kcal/mol. Results revealed that the compound (**3**) bound to the anionic substrate binding site of AChE and would act as a competitive inhibitor (Figure 3C) of the enzyme. Isopropyl group in the side chain at carbonyl group-containing C-2′ position mediated π-alkyl interaction with Phe330. In addition, butyric acid ester group present in the compound (**3**) at C-1′ position mediated alkyl-alkyl interaction with Trp84 and Leu127 amino acid residues further increased the binding.

**FIGURE 4:**
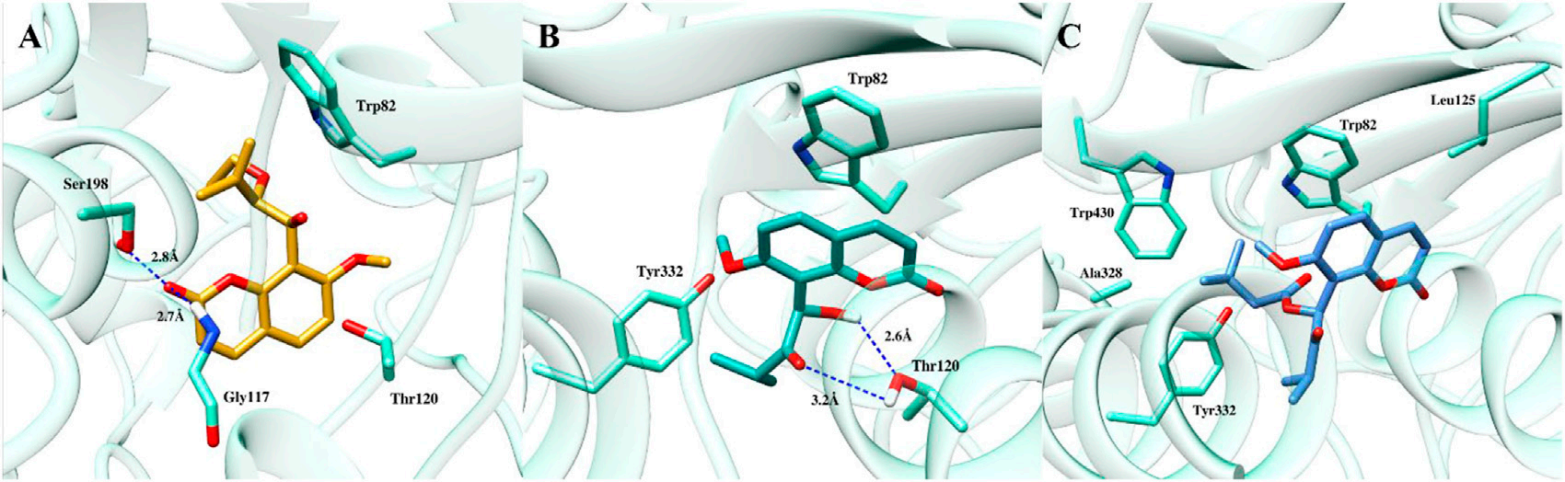
Docked pose of all the coumarin derivatives with BChE. **(A)** 2′-*O*-ethylmurrangatin (**1**), **(B)** murranganone (**2**) and **(C)** paniculatin (**3**) resided in the active site of BChE.

At the binding site of BChE enzyme, compound (**1**) resided into the aromatic gorge by establishing hydrophobic and hydrogen bond interactions with moderate binding affinity of −6.47 kcal/mol (Figure 4A). Carbonyl group at C-2 position mediated hydrogen bonding with Gly117 and Ser198. Similarly, methylene group in the side chain of C-8 bent towards the Trp82, while the coumarin ring was observed to be bent towards the Thr120. The docked pose of compound (**2**) at the binding site of BChE demonstrated two hydrogen bonds with the highest binding affinity of −7.92 kcal/mol (Figure 4B). Hydroxyl group at C-1′ and ketonic group at C-2′ mediated hydrogen bonds with Thr120, whereas the coumarin ring stacked against Trp82 residue. Furthermore, isopropyl group present in the side chain of ketonic group at C-2′ position mediated hydrophobic interaction with Tyr332. The potency of compound (**2**) against the enzyme BChE can be attributed, in part, to the two hydrogen bonds with the Thr120 and π–π stacking with Trp82 residue. The third coumarin paniculatin (**3**) presented hydrophobic interactions only at the binding site of BChE with good binding affinity of −7.01 kcal/mol (Figure 4C). The ester group at C-1′ position also mediated hydrophobic interactions with Ala328, Tyr332, and Trp430, whereas the coumarin ring interacted with Trp82 by mediating hydrophobic interaction.

The docking studies revealed that 2′-*O*-ethylmurrangatin (**1**) which has ethoxy group at C-2′, unlike murranganone (**2**) and paniculatin (**3**) having ketonic group at C-2′, did not inhibit AChE and was also a weak inhibitor of BChE. Paniculatin (**3**) closely resembles murranganone (**2**) with the only difference being the presence of a large butyric acid ester group in compound (**3**) at C-1′ instead of hydroxyl group in compound (**2**). The data suggested that the ketone functionality at C-2′ might be an important structural determinant of cholinesterase inhibition. Also, the isopropyl group in compounds (**2**) and (**3**) mediated the hydrophobic, alkyl-alkyl and π-alkyl interactions with aromatic amino acids such as tyrosine, tryptophan and phenylalanine showing importance of this group in binding to the active sites of AChE and BChE proteins. The absence of isopropyl group in compound (**1**) decreased the interaction and thereby activity against AChE.

### 3.3 Molecular dynamics (MD) simulation

**FIGURE 5:**
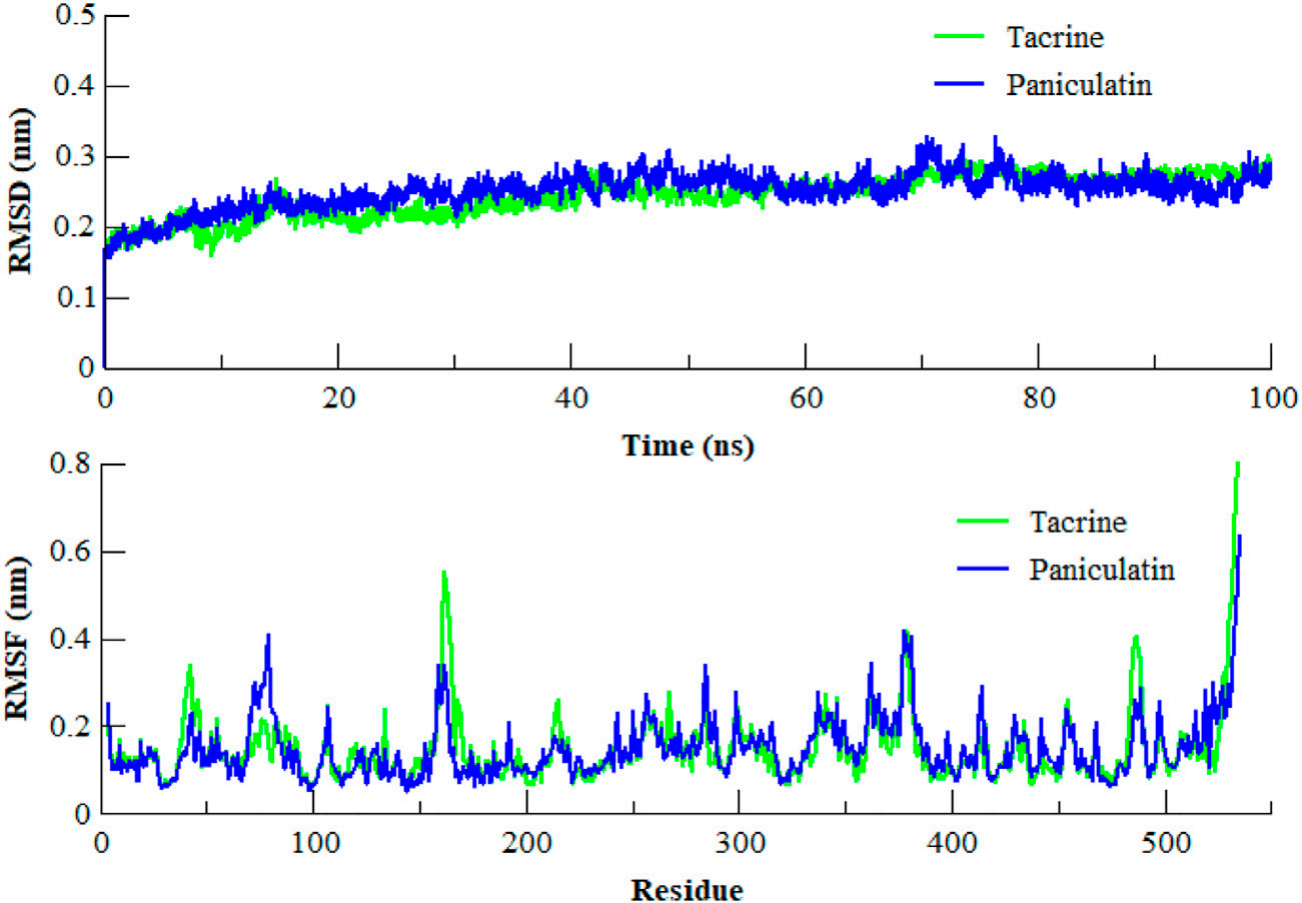
RMSD and RMSF of the backbone atoms (CA, N, and C) of AChE in complex with Paniculatin (**3**) and Tacrine with respect to the initial structure as a function of time. RMSD of Tacrine shown in green color while Paniculatin (**3**) shown in blue color.

The MD simulation is a computational technique which provides the information about dynamic behavior of a molecular system (Hollingsworth and Dror, 2018). It is also useful in determining the time-dependent stability of protein-ligand complexes. To determine the most persistent interactions and to assess the stability of protein-ligand complexes over time, MD simulation studies were carried out for the coumarins. We employed 100 ns MD simulation of AChE in complex with the most potent compound paniculatin (**3**) and the standard drug Tacrine (Figure 5). During the course of simulation, the coumarin ring of paniculatin (**3**) helped in stacking between the Trp84 and Phe330 residues. The characteristic hydrophobic contacts of the compound with Leu127, Ile439, and Tyr442 residues were found to be persistent during simulation. Results of MD simulation testified the docking results since most of the docking interactions were shown to be consistent during the course of simulation. The root mean square deviation (RMSD) of the backbone atoms was plotted to determine the stability of the complex. As evident from Figure 5, the stability of AChE in complex with paniculatin (**3**) was very much comparable to Tacrine-AChE complex during the simulation. Both complexes showed RMSD of less than 0.32 nm with inconsiderable fluctuations. Similarly, both complexes showed similar patterns of root mean square fluctuation (RMSF) values and the average RMSF for both the complexes was found to be 0.14 nm. It was observed that the anionic site residues were more rigid and less flexible during the course of simulation. These observations indicated that paniculatin (**3**) significantly anchored in the active aromatic gorge of AChE and contributed to the stability of protein which pronounced the observed biological activity.

### 3.4 *In silico* absorption, distribution, metabolism and excretion (ADME) prediction

**FIGURE 6:**
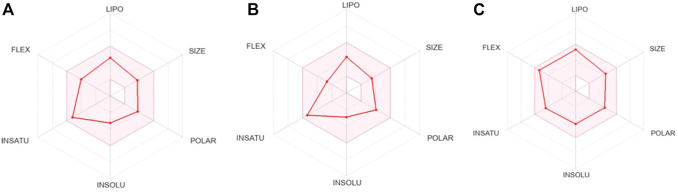
Bioavailability radar of **(A)** 2′-*O*-ethylmurrangatin (**1**), **(B)** murranganone (**2**) and **(C)** paniculatin (**3**).

Most of the drug candidates fail during the clinical trials owing to their insufficient efficacy and/or toxicity. Apart from efficacy and toxicity, other important parameters leading to failure in drug development are pharmacokinetics and bioavailability (Thompson, 2000; Hassan et al., 2022). *In silico* ADME studies is an alternative to experimental techniques in the early process of drug discovery with an aim to increase the success rate in clinical trials. Herein, SwissADME was used to predict and evaluate the ADME profile of the tested coumarin derivatives and the results obtained are summarized in Table 2. To evaluate the oral bioavailability, a radar was plotted presenting six physiochemical properties including liphophilicity, size, polarity, solubility, saturation and flexibility. As evident from Figure 6, all six physiochemical chemical properties for the three tested compounds (**1–3**) fall into the acceptable range (pink region) and all the compounds were in significant agreement with the given criteria to be considered as drug-like.

**TABLE 2:** Predicted ADME profile of tested coumarin derivatives (**1–3**).

*Ligands*	*Mol*	*HBA*	*HBD*	*TPSA* (*Å* ^*2*^)	*iLOGP*	*WLOGP*	*GI absorption*	*BBB*	*P-gp substrate*	*Lipinski violations*
	*Weight (g/mol)*									
***1***	304.34	5	1	68.90	3.00	2.49	High	Yes	No	0
***2***	276.28	5	1	76.74	1.97	1.74	High	No	No	0
***3***	360.40	6	0	82.81	3.31	3.33	High	No	No	0

**FIGURE 7:**
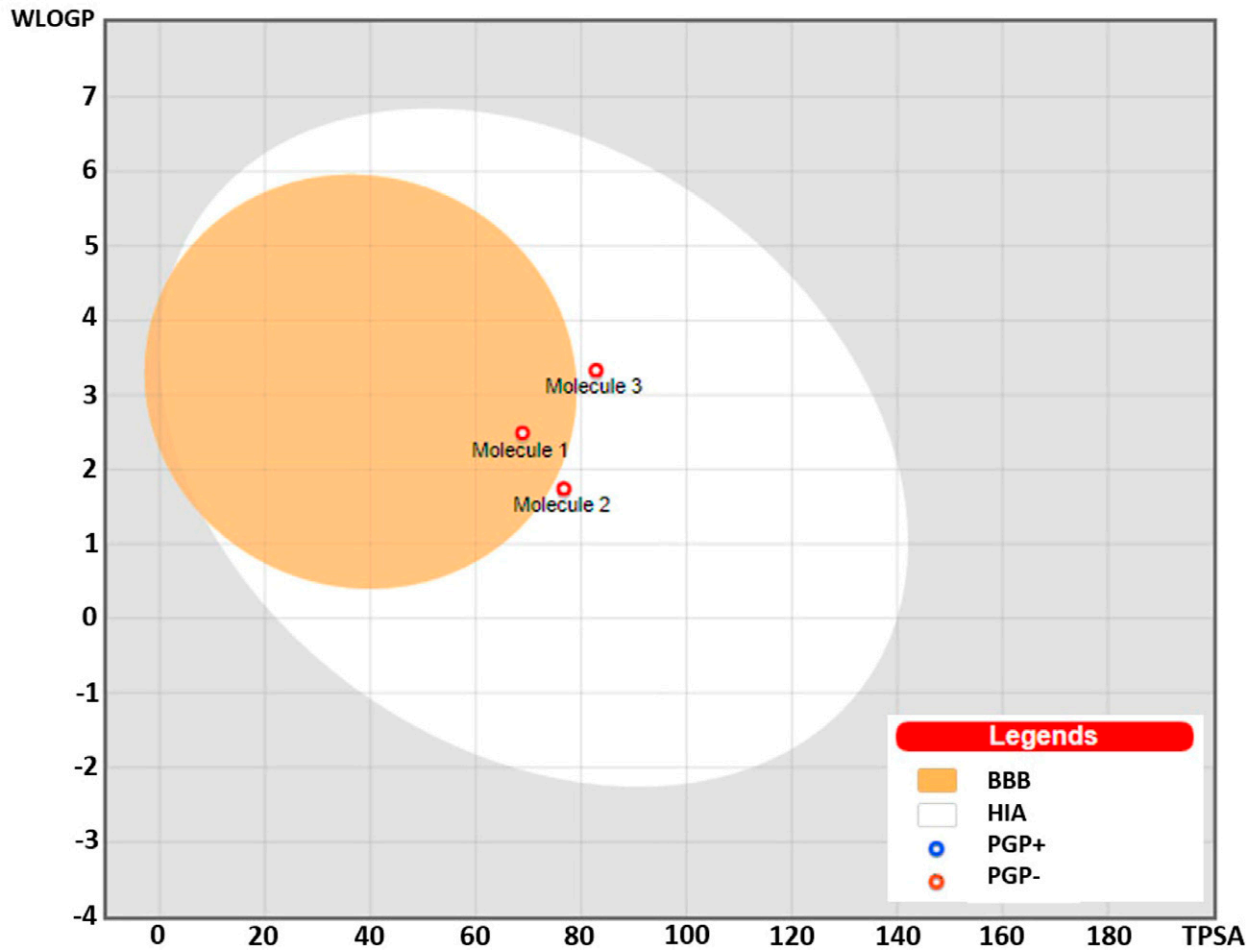
BOILED-Egg predictive model showing placement of compounds (**1**), (**2**), and (**3**). Compound (**1**) was placed in the yolk suggesting probability for permeability to the brain, while compounds (**2**) and (**3**) were placed in the white region predicting high gastrointestinal absorption. The red dots predicted to be not effluated from the central nervous system (CNS) by the P-glycoprotein.

The Brain or Intestinal Estimated Permeation predictive model (BOILED-Egg) was applied to estimate the gastrointestinal absorption and permeability to brain by computing the lipophilicity and polarity of the molecules. The WLOGP values (Log P calculated using Wildman and Crippen method) and topological polar surface area (TPSA) were estimated and placed in the BOILED-Egg predictive model. As evident in Figure 7, compound (**1**) was placed in the yolk (yellow) region of the egg suggesting high probability of this compound to cross blood-brain barrier (BBB) and permeate to the brain, while compound (**2**) and (**3**) fall into the white region of the egg indicating high probability of these compounds to be absorbed in the gastrointestinal region. The red dots in the figure suggest that these compounds are not predicted to be effluated from the central nervous system (CNS) by the P-glycoprotein (P-gp).

## 3 Conclusion

The 8-substituted-7-methoxy coumarins isolated from orange jasmine were identified as a new class of natural coumarins active against the cholinesterases enzymes; thereby can be regarded as potential candidates for AD. These coumarins showed non-selective moderate to good *in vitro* activity against both AChE and BChE by mixed-type inhibition mechanism. The structural features important for binding to the receptors were identified using molecular docking technique. It was observed that the hydrogen bonding by ketone and hydroxyl functionalities at different positions played an important part in binding to the receptor, whereas the isopropyl group at the C-3′ position was involved in the non-covalent hydrophobic interactions. The stability of drug-enzyme complex for the most active compound (**3**) as measured using the MD simulation studies was comparable to the standard drug Tacrine. These compounds showed optimal physicochemical properties to be regarded as drug-like molecules and can be explored further as lead molecules in order to improve the binding and efficacy.

## Data Availability

The raw data supporting the conclusion of this article will be made available by the authors, without undue reservation.
